# Exploratory analysis of neutrophil extracellular traps in synovial fluid and plasma from children with juvenile idiopathic arthritis

**DOI:** 10.3389/fped.2026.1833576

**Published:** 2026-06-24

**Authors:** Lillemor Berntson, Caroline Sandström, Andreas Elfving, Fariborz Mobarrez

**Affiliations:** 1 Department of Women’s and Children’s Health, Uppsala University, Uppsala, Sweden; 2 Department of Medical Sciences, Clinical Chemistry, Uppsala University, Uppsala, Sweden

**Keywords:** extracellular vesicles, juvenile idiopathic arthritis, myeloperoxidase, neutrophil extracellular traps, neutrophils, platelets, synovial fluid

## Abstract

**Background:**

The innate immune system is increasingly recognised as a key driver in the pathogenesis of juvenile idiopathic arthritis (JIA). Extracellular vesicles (EVs) have emerged as important mediators of inflammation, potentially reflecting cellular activation and disease processes *in vivo*. The aim of this exploratory pilot study was to investigate the presence of neutrophil- and platelet-derived EVs in the plasma and synovial fluid of children with JIA and compare these with plasma levels in healthy controls.

**Methods:**

Seven children with active, untreated JIA and ten healthy controls were included. Citrate plasma and synovial fluid were analysed for neutrophil- and platelet-derived EVs using flow cytometry. Disease activity was assessed using the Juvenile Arthritis Disease Activity Score (JADAS27) and functional impairment via the Childhood Health Assessment Questionnaire (CHAQ). Non-parametric tests were used in analyses.

**Results:**

Neutrophil- and platelet-associated EVs were detectable not only in plasma but also in synovial fluid in all children with JIA. Plasma levels of CD41+ EVs and MPO+ EVs were significantly higher in the JIA group compared with controls. Activated platelet EVs (CD41+ and CD41 + CD62P) were detected predominantly in plasma and only at low levels in synovial fluid. MPO+ and H3Cit+ EVs were present in both compartments, indicating neutrophil activation and NET-related components. No correlations between EV levels and clinical or laboratory variables were found.

**Conclusion:**

This exploratory pilot study is the first to assess platelet- and neutrophil/NET-associated EV phenotypes in both plasma and synovial fluid in children with JIA. The results indicate systemic platelet activation and NET-related activity in both circulation and the inflamed joint compartment.

## 1 Introduction

Juvenile idiopathic arthritis (JIA) is the most common paediatric rheumatological disease and can be classified into seven categories based on criteria defined by the International League of Associations for Rheumatology (ILAR) (1).

The pathophysiology is complex and has primarily been considered a dysregulation of the adaptive immune system, but increasing evidence also suggests the involvement of the innate immune system in the pathogenesis (2, 3). The inflammatory response is mediated by complex interactions between immune cells such as lymphocytes, monocytes, macrophages, platelets and neutrophils (4, 5).

As key effectors of the innate immune system, neutrophils play an essential role in immune defence. Earlier studies on JIA have shown an upregulation of the neutrophilic genes suggesting an activated state in neutrophils even during disease quiescence (6). Neutrophilic transcriptomes have also shown considerable specificity (3). In addition to their unique antimicrobial functions, neutrophils also play a central role in driving inflammation by releasing pro-inflammatory mediators.

Further support for the role of neutrophil granulocytes in the disease process of JIA is the release of myeloperoxidase (MPO), an enzyme secreted from primary granules in neutrophils. In the setting of sterile inflammation, MPO and MPO-derived oxidants are considered pathogenic, promoting inflammation and causing tissue damage (7). MPO levels have been shown to be increased in plasma of children with JIA as well as in adults with rheumatoid arthritis, compared to healthy controls (8, 9), in adults also in the synovial fluid (10).

Within the inflammatory milieu, neutrophil granulocytes produce web-like extracellular structures termed neutrophil extracellular traps (NETs). These are composed of DNA fibres and proteins such as histones and MPO. NETs can affect the immune system in multiple ways and have therefore attracted attention in the context of autoimmune diseases, where they are found to initiate and perpetuate inflammation (11, 12).

In one study on NETs in JIA, increased NETs formation in peripheral blood compared to healthy controls was found (13). Another study showed that neutrophils isolated from patients with oligo JIA, poly JIA and ERA generated more NETs than neutrophils from controls (14). The increase in NETs was associated with disease activity in both studies.

NETs can originate from cells other than neutrophils. Recent studies reveal an active role of platelets in modulating inflammation, for instance by forming platelet-neutrophil aggregates (PNAs) which can release proinflammatory mediators and NETs, as well as trigger neutrophil recruitment and migration. One study has shown that children with JIA have higher levels of PNAs compared to controls and that these PNAs have elevated CD62P-expression, a marker of platelet activation (15).

Neutrophils are the most common white blood cell in the synovial fluid (SF) of patients with JIA, accounting for approximately 60% of cells (16). Synovial fluid neutrophils from children with oligoarticular and polyarticular JIA have shown an activated and aged phenotype compared to peripheral blood neutrophils (17, 18). In one study on JIA, SF neutrophils expressed HLA-DR, suggesting antigen-presenting properties and hence the possibility of interacting with the adaptive immune system (17). Furthermore, synovial fluid neutrophils have shown a decrease in phagocytic capacity compared to peripheral blood neutrophils which may contribute to sustained inflammation (18).

Taken together, these findings support a prominent role of neutrophils and platelets in JIA, both systemically and within the inflamed joint. However, many of these processes are dynamic and compartment-dependent, and soluble markers or cell counts alone may not fully capture ongoing cellular activation at the time of sampling. A complementary approach is therefore to analyse extracellular vesicles (EVs), which are released during cellular activation and can be phenotyped to reflect cell-of-origin and activation states *in vivo*. EVs are membranous particles released by all cell types, carrying proteins, lipids and nucleic acids, regulating biological processes through cellular signalling. Both the surface markers of the vesicle and its cargo reflect the cellular origin of the EV, potentially providing a picture of the inflammatory state of the donor cell at the time of EV release (19). One study that characterised the protein profiles of EVs from plasma and synovial fluid from oligo JIA patients found differing profiles between synovial fluid EVs and plasma EVs. In another study from the same group, differences in the microRNA expression in EVs from synovial fluid and plasma from oligo JIA patients were found (20, 21). Increased levels of EVs in plasma derived from activated platelets have been found in adults with RA and in one study of children with JIA (22, 23).

Assessment of EVs expressing MPO or citrullinated histone H3 (H3Cit) may reflect neutrophil activation and NET-related processes. H3Cit is considered a relatively NET-specific marker, and circulating H3Cit has been shown to increase during human endotoxemia. Importantly, H3Cit can be detected bound to microvesicles by flow cytometry, supporting EV-associated detection of NET components *in vivo* (24).

The protein CD41 is expressed by platelets, and EVs positive for CD41 are considered to originate from platelets (23). CD62P (*P*-selectin) is a cell adhesion molecule expressed by activated platelets (25) and EVs expressing CD41 together with CD62P support activated platelet origin. To further explore possible inflammation in which mainly platelets are involved, EVs expressing both CD41 and CD40L can be studied. CD40L is expressed on both platelets and activated T-cells but is mostly considered an indicator of active inflammation (26).

Current evidence indicates altered EV profiles in JIA, but studies assessing neutrophil- and platelet-associated EVs simultaneously in paired plasma and synovial fluid are lacking. We aimed to address this gap by analysing EVs associated with neutrophil activation/NET formation and platelet activation in both compartments, and by exploring their associations with clinical measures.

## 2 Material and methods

### 2.1 Study participants

Seven children diagnosed with JIA and classified according to the ILAR criteria (1) were included at the Unit of Paediatric Rheumatology, Uppsala University Hospital, between 2021 and 2025. Disease activity was assessed using the Juvenile Arthritis Disease Activity Score (JADAS27). This composite score comprises a joint count (0–27 active joints), patient-reported global assessment of well-being on a visual analogue scale (VAS) (0–10 cm), assessed by a parent if the child is ≤9 years old, physician's global assessment of disease activity on VAS (0–10 cm) and normalised erythrocyte sedimentation rate {[E-SR (mm/h) −20]/10} to a scale (0–10). The maximum total score of JADAS27 is 57 (27). Functional impairment was assessed using the Child Health Assessment Questionnaire (CHAQ) (0–3) (28). Ten healthy, age- and sex-matched controls were also included in the study. Exclusion criteria included the presence of any inflammatory disease, diabetes, any atopic disease with continuous medication or special diet because of intolerance. The study design and participant flow are summarized in Supplementary Figure S1.

### 2.2 Sample collection and handling

Blood and synovial fluid samples were collected simultaneously at disease onset or in one case during a disease flare after ten months of no symptoms and no medical treatment. Blood samples were collected for routine laboratory testing as well as flow cytometric testing. Citrate plasma samples were handled equally in participants and controls, drawn into citrate-coated tubes for flow cytometric analysis. Synovial fluid samples were drawn from the affected joint just before intraarticular corticosteroids were given. Plasma and synovial fluid samples were processed and frozen within 60 min of withdrawal. They were centrifuged at 1,500 × g for 20 min and then frozen at −70 ℃. Blood samples for routine laboratory testing were analysed by the clinical chemistry unit. Samples from controls were collected pre-operatively from children admitted for minor surgery, who were otherwise healthy and on no medication for any disease.

### 2.3 Flow cytometric measurement of EVs

The frozen plasma and synovial fluid (SF) samples were thawed in a 37 ℃ water bath for 5 min. Samples were then centrifuged at 2,000 × g for 20 min at room temperature (RT). To obtain an EV-enriched pellet the supernatant was transferred to new tubes and centrifuged at 20,000 × g for 45 min at RT. The supernatant was discarded, and the remaining EV-enriched pellet was used for flow cytometric analysis. Twenty µL of sample from the pellet was transferred to a 96-well plate containing 5 μL of conjugated antibodies. Samples were stained in separate panels to avoid spectral overlap: panel 1 included CD41-FITC, CD62P-PE and CD40L-APC; panel 2 included H3Cit-Alexa Fluor 488 and MPO-APC (Beckman Coulter, Brea, CA, USA and Abcam, Cambridge, UK). Plasma from the control group were only analysed for CD41 and MPO. The plate was incubated in the dark for 20 min before adding 120 μL of CytoFLEX sheath fluid. EVs were measured using the CytoFLEX flow cytometer. The EV gate was determined using Spherotech Nano fluorescent Yellow Particles of sizes 0.22 μm, 0.45 μm, 0.88 μm & 1.35 μm (Spherotech, Lake Forest, IL, USA). EVs were defined as vesicles that were between 0.1 and 1 μm and positive for antibodies. Results are presented as the number of EVs per microliter of plasma or SF.

### 2.4 Statistical analysis

A *p*-value of <0.05 was considered significant and non-parametric tests were used in analyses. Demographic data were expressed using frequencies and percentages for categorical variables, and median with interquartile range (IQR) for continuous variables. For comparison between groups the Chi-square test and Mann–Whitney *U*-test were used. Spearman's rank order correlation was used to analyse correlations between variables. Analyses were performed using the Statistical Package for Social Sciences version 28 (SPSS Inc., Illinois, USA) and JMP software (SAS Institute, v17.0, Cary, North Carolina, USA). GraphPad Prism (10.0, GraphPad Software Inc, La Jolla, CA) was used to create figures.

### 2.5 Ethics approval and consent to participate

The study was approved by the regional ethics committee in Uppsala County (reference 2006/327; 2006/327/1; 2006/327/3; 2014/335) and from the Swedish Ethical Review Authority (Dnr 2021-01614; 2024-01478-02). Verbal informed consent was obtained from parents and children. Written informed consent was obtained from children at the age of twelve or older and from parents.

## 3 Results

### 3.1 Clinical assessments of participants

Seven children with JIA and ten healthy controls were included in the study. Table 1 shows the demographic data of patients and controls. Six patients were classified as oligoarticular JIA, and one as ERA. The boy with ERA exhibited an oligoarticular pattern, with arthritis in one knee joint but no signs of sacroiliitis; however, he had a family history of ankylosing spondylitis in a first-degree relative and onset of disease above six years of age. Six of the seven patients were included during the first visit after onset at the paediatric ward, with a disease duration of median 0.1 years (IQR 0.1–0.2) with one active knee joint. One patient had a disease duration of 13.7 years at time of inclusion, after a period of no symptoms and no medication for ten months. This patient also had an oligoarticular disease course and one active knee joint at inclusion. All participants were without medical treatment at time of inclusion. The gender and age distribution did not differ significantly between patients and controls (*p* = 0.486 and *p* = 0.626 respectively).

**Table 1:** Demographic data in seven children with juvenile idiopathic arthritis (JIA) and the ten healthy controls.

Md (IQR)	Total group JIA, *n* = 7	Controls, *n* = 10	*p*-value	
Gender F/M (%)	3/4 (42.9/57.1)	6/4 (60/40)	0.486*	
Age at onset, years	6.6 (2.1–13.8)			
Age at inclusion, years	11.7 (2.7–14.4)	9 (6.2–12.8)	0.626**	
Disease duration, years	0.1 (0.1–0.2), 13.7^a^			
JADAS27 (0–57)	10.6 (4.2–17.1)			
CHAQ (0–3)	0.75 (0.38–1)			
E-SR mm/h	25 (11–66), 9 – 69^b^			
Leukocytes 10^9^/L	6.8 (5.2–8.0)			
Neutrophils 10^9^/L	3.4 (2.8–4.4)			
Platelets 10^9^/L	321 (310–503)			
Individual presentation of the seven children with JIA				
ILAR category	Gender F/M	Age at onset (years)	Age at inclusion (years)	Disease duration (years)
ERA (Oligoarticular)	M	11.6	11.7	0.1
Oligoarticular	F	14.2	14.4	0.2
Oligoarticular	M	1.3	1.4	0.1
Oligoarticular	F	13.8	13.9	0.1
Oligoarticular	M	6.6	6.7	0.1
Oligoarticular pers	M	2.1	15.8	13.7^a^
Oligoarticular	F	2.5	2.7	0.2

F, female; M, male; Md, median; IQR, interquartile range; HAQ, childhood health assessment questionnaire; E-SR, erythrocyte sedimentation rate; ILAR, international league of associations for rheumatology; ERA, enthesitis-related arthritis; Oligoarticular pers, Oligoarticular persistent.

* Pearson Chi-Square test.

** Mann–Whitney *U*-test.

a One patient was included during a disease flare after having been without medical treatment for ten months.

b min-max.

### 3.2 Levels of extracellular vesicles

**Figure 1:**
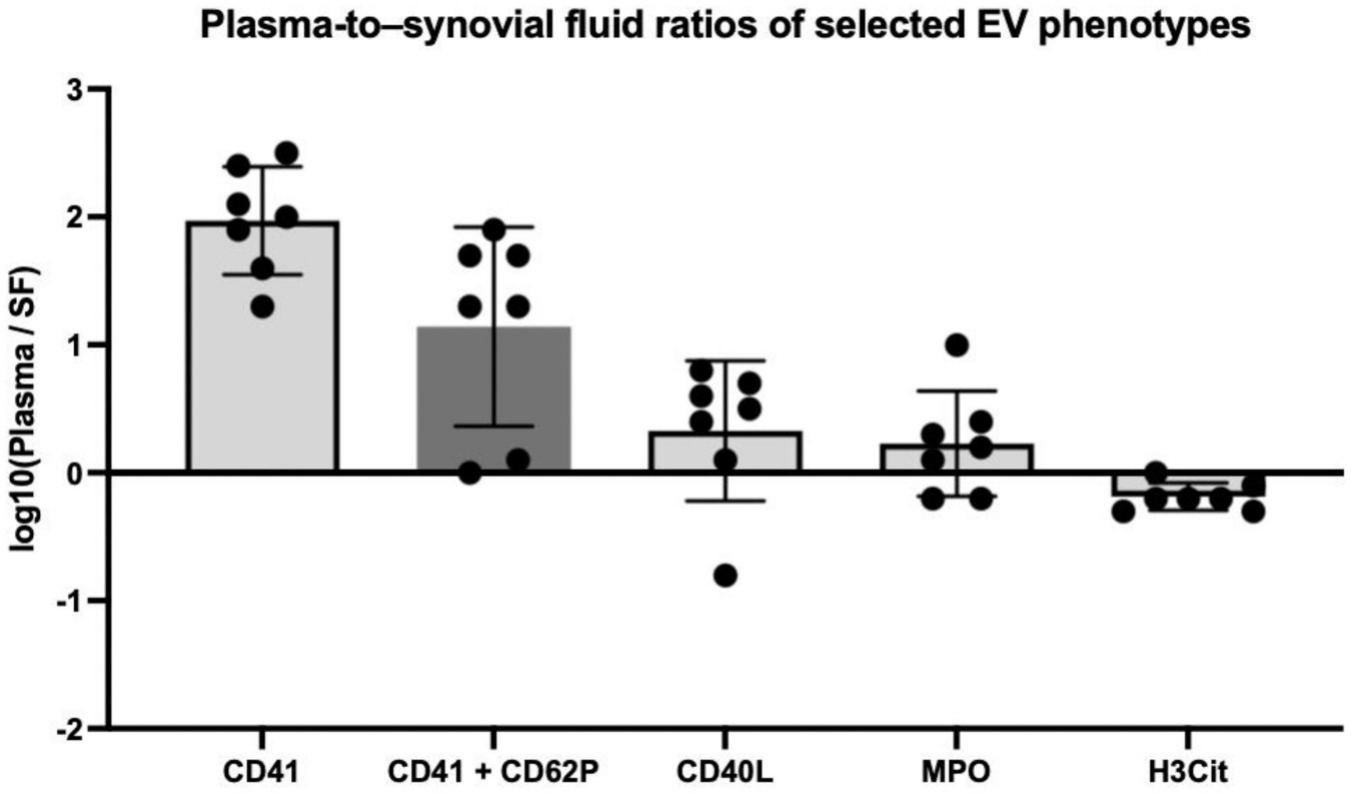
Relative compartment distribution of extracellular vesicle phenotypes. Log10-transformed plasma-to–synovial fluid ratios are shown for CD41-, CD41 + CD62P-, CD40L-, MPO-, and H3Cit-positive EVs. Each dot represents one individual. Values above zero indicate relative predominance in plasma, whereas values below zero indicate relative enrichment in synovial fluid.

**Figure 2:**
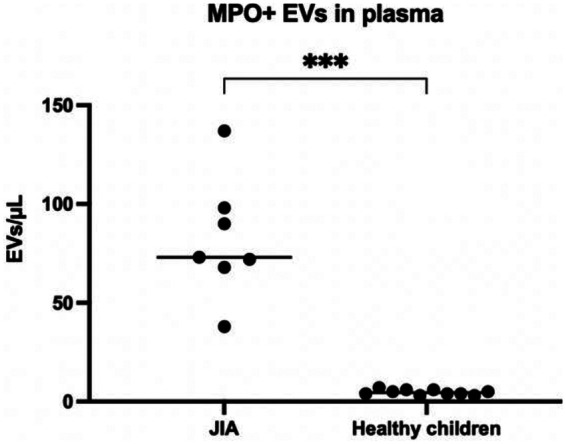
Plasma levels of MPO+ extracellular vesicles (EVs/µL) in children with juvenile idiopathic arthritis (JIA, *n* = 7) and healthy controls (*n* = 10). Each dot represents one individual; horizontal lines indicate medians. ***p < 0.001, Mann–Whitney *U*-test.

EV levels and phenotypes in plasma and synovial fluid from children with JIA, together with plasma levels in controls, are summarised in Table 2. To highlight compartment distribution, plasma-to-synovial fluid ratios were calculated and log10-transformed (Figure 1). Overall, compartmentalization differed by phenotype, with a strong plasma predominance for platelet-derived EVs (CD41+), intermediate patterns for MPO- and CD40L-positive EVs, and synovial enrichment for H3Cit-positive EVs (Figure 1). Platelet-derived EVs (CD41+) were detected in both compartments but at markedly lower levels in synovial fluid, and EVs reflecting platelet activation (CD41 + CD62P+) were predominantly observed in plasma with only low levels in synovial fluid. In contrast, CD41 + CD40L+ EVs were not detected in synovial fluid, whereas neutrophil/NET-associated phenotypes (MPO, H3Cit) were readily detected in both compartments (Table 2). In addition, plasma levels of CD41-positive EVs and MPO-positive EVs were higher in the JIA group compared with controls (Table 2; *p* = 0.025 and *p* < 0.001, respectively; Figure 2). No significant correlations were found between levels of CHAQ or JADAS27 and levels of EVs in plasma or synovial fluid (data not shown).

**Table 2:** Levels of extracellular vesicles in plasma and synovial fluid in seven patients with juvenile idiopathic arthritis (JIA) and ten healthy children.

Target Md (IQR)	JIA *N* = 7		Healthy children *N* = 10	*p*-value
	Plasma (EVs/µL)	Synovial fluid (EVs/µL)	Plasma (EVs/µL)	
CD41+	784 (554–1,736)	9 (7–15)	487 (361–530)	0.025*
CD41 + CD40L	38 (15–90)	0	−	
CD41 + CD62P	112 (39–210)	3 (2–7)	−	
CD40L+	20 (15–27)	7 (7–11)	−	
MPO+	73 (68–98)	50 (39–64)	5 (4–6)	<0.001*
H3Cit+	5 (5–6)	8 (7–9)	−	

Md, median; *IQR*, interquartile range; EV, extracellular vesicle; CD, cluster of differentiation; CD41, platelets; CD41+CD40L, T-cells or platelets; CD41+CD62P. activated platelets; CD40L, T-cells or platelets; MPO, myeloperoxidase; H3Cit, citrullinated histone H3.

* Mann–Whitney *U*-test was used to compare plasma levels between the JIA group and control group.

## 4 Discussion

To our knowledge, this is the first study to characterise extracellular vesicle phenotypes related to platelets and neutrophil granulocytes in synovial fluid from children with juvenile idiopathic arthritis. By analysing paired plasma and synovial fluid samples, we were able to directly explore compartment-specific EV signatures in active disease.

Recent studies indicate that platelets actively participate in the pathogenesis of JIA, rather than serving only as passive bystanders (5, 15). We observed significantly higher levels of platelet-derived EVs (CD41+ EVs) in plasma of children with JIA compared to controls, in line with increased levels of CD41+ EVs in high disease activity, as shown by Kumar et al. (23). Furthermore, our data demonstrated increased levels of CD41 + CD62P EVs in plasma, consistent with systemic platelet activation; however, this analysis did not include a comparison with controls. We also detected CD41+ and CD41 + CD62P EVs in synovial fluid, albeit at lower levels. This pattern may reflect primarily systemic platelet activation rather than extensive local shedding into the joint space, but matrix-related factors (e.g., viscosity, debris, and pellet recovery) could also influence synovial EV quantification and should be addressed in future studies using standardised pre-analytical handling and recovery controls.

In our study, we observed significantly higher plasma levels of MPO+ EVs in the JIA group than in controls, consistent with prior studies on increased levels of MPO in plasma in children with JIA (8, 29). MPO is primarily released by neutrophils but can also be released by monocytes (7). Together with the finding of citrullinated histone H3 (H3Cit+) EVs in plasma these findings suggest activation of peripheral neutrophils in JIA, also in agreement with earlier studies (6, 15).

Additionally, we detected relatively high levels of MPO+ EVs in the synovial fluid of inflamed joints which has been described earlier in adults with RA but not in children with JIA (10). The presence of MPO is associated with the production of reactive oxygen species (ROS) and cytokines, supporting the chronic inflammatory state (30). The finding of MPO+ EVs, together with the presence of H3Cit+ EVs in synovial fluid, suggests neutrophil activation within the joint microenvironment and aligns with previous observations of activated/aged synovial neutrophil phenotypes in JIA (17, 18). Importantly, the paired sampling design strengthens the interpretation that the joint compartment carries a distinct inflammatory EV fingerprint rather than being a diluted reflection of plasma.

The presence of H3Cit+ EVs in both plasma and synovial fluid, together with MPO+ EVs, is compatible with increased neutrophil activation and NET-associated processes in JIA, as H3Cit and MPO are commonly linked to NET biology. Earlier studies have shown an augmented NET formation and increased levels of NET-derived products in the blood of patients with JIA (13–15). Our results support these findings and suggest that NET-related activity may also be prominent in the articular compartment during active disease. NETs may serve as autoantigens and stimulate proinflammatory responses in fibroblast-like synoviocytes, cells known to invade cartilage in RA, thereby contributing to joint damage (31). Synovial fibroblasts in JIA have been found more invasive and cartilage degrading than synovial fibroblasts in healthy children (32), and may promote neutrophil recruitment and prolonged neutrophil survival (33–35). These interactions could contribute to sustained inflammation, leukocyte infiltration, and cartilage degradation.

The cellular interaction between neutrophils and platelets is another factor in inflammation. As Parackova et al. (15) demonstrated, activated platelets form platelet-neutrophil aggregates (PNAs) in plasma in children with JIA and this contributed to neutrophil activation and pro-inflammatory properties. Platelets are capable of secreting various cytokines and chemokines and expressing Toll-like receptors and cell adhesion molecules, allowing interaction with other immune cells, mainly neutrophils and monocytes (36). Since we detected EVs suggesting the presence of activated platelets in synovial fluid, as well as neutrophils, we speculate that PNA formation also occurs in the synovial fluid of children with JIA. In addition to formation of PNAs, both platelets and platelet-derived EVs can interact with neutrophils to initiate neutrophil extracellular trap (NET) formation and the release of MPO, potentially increasing inflammation (25). Whether this is the case in JIA remains to be elucidated and further research is needed to clarify the role of platelets and platelet-derived EVs in JIA.

Our study has several limitations; one is the small number of participants but all of them were without treatment at study inclusion and all but one belonged to the same JIA category. Another limitation could be that control plasma was only analysed for EVs expressing CD41 and MPO, limiting comparisons and conclusions. Given the exploratory nature and the limited sample size, limiting a possibility for sample size calculation, the results should be interpreted as hypothesis-generating. Comparisons between plasma and synovial fluid may be influenced by matrix-related factors and pellet recovery, which warrants future inclusion of recovery controls and standardised handling across compartments. Another possible weakness was the wide spread in age of participants since recent studies on synovial biopsies reveal that younger children have more plasma cells in their synovium compared to older children (37). Whether this finding has implications for our results remains uncertain.

## 5 Conclusion

We found evidence of NET-related EV signatures not only in plasma but also in the articular compartment of children with JIA. Our data suggest that systemic platelet activation signals are primarily reflected in circulation, while local joint inflammation is more strongly mirrored by NET-related EV signatures. The parallel analysis of paired samples reinforces the notion that the joint harbours a compartment-specific inflammatory EV profile distinct from that observed in circulation. With the aim of developing targeted treatment approaches in the management of JIA further exploration of the pathophysiological mechanisms operating both systemically and locally is warranted. Despite the limited sample size, these pilot data provide a novel basis for future large-scale stratification studies in JIA.

## Data Availability

The raw data supporting the conclusions of this article will be made available by the authors, without undue reservation.
