# Supplementary material for: Exploratory analysis of neutrophil extracellular traps in synovial fluid and plasma from children with juvenile idiopathic arthritis
Source: Front Pediatr. 2026 Jun 24;14:1833576. doi: 10.3389/fped.2026.1833576 (PMC13341427; doi:10.3389/fped.2026.1833576)
Supplement: Supplementary file 1 [file Image1.pdf]

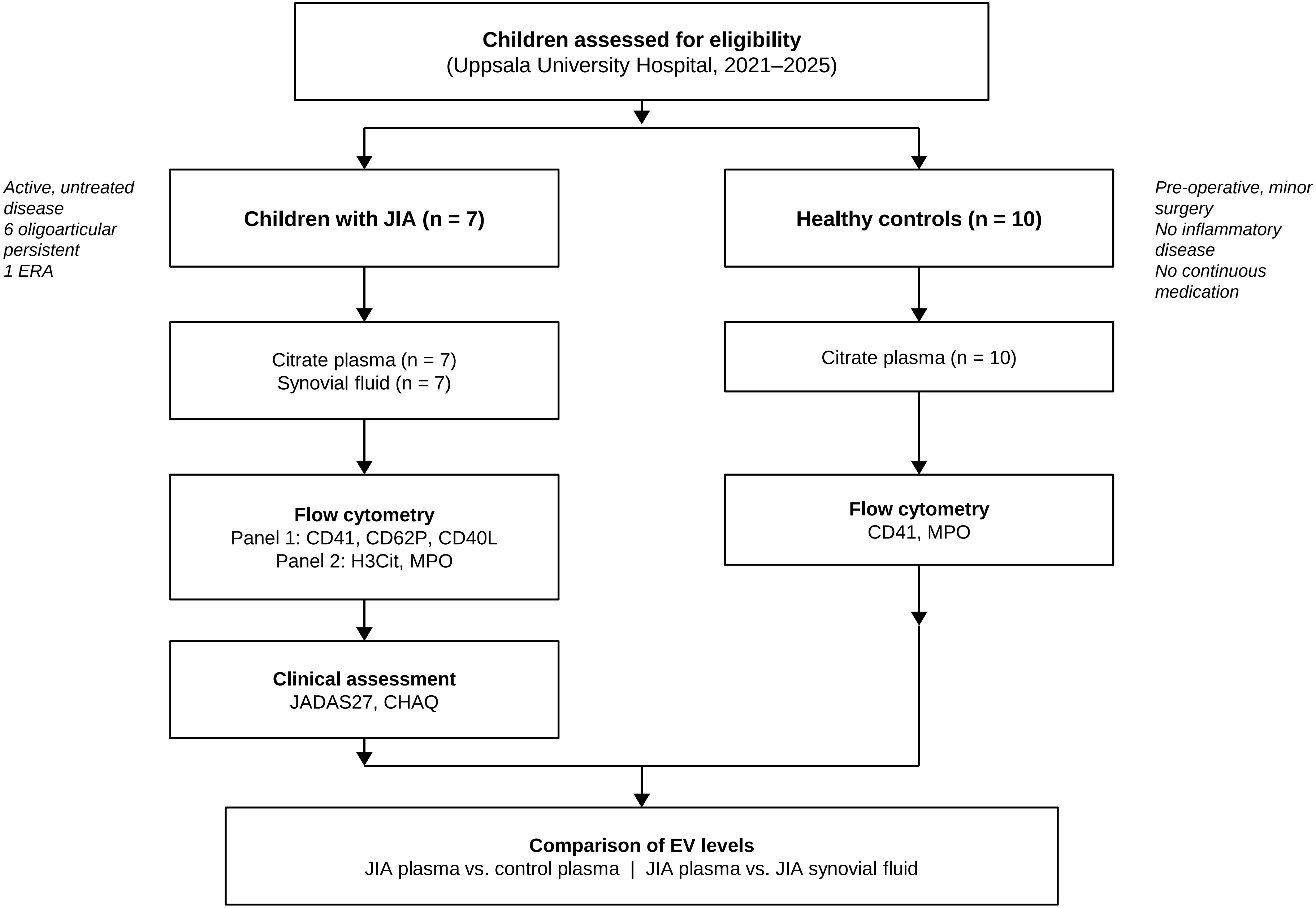

Supplementary Figure 1. Study flowchart illustrating participant selection, sample collection, and analytical procedures.
